# GPS-based fine-scale mapping surveys for schistosomiasis assessment: a practical introduction and documentation of field implementation

**DOI:** 10.1186/s40249-021-00928-y

**Published:** 2022-01-15

**Authors:** Lydia Trippler, Mohammed Nassor Ali, Shaali Makame Ame, Said Mohammed Ali, Fatma Kabole, Jan Hattendorf, Stefanie Knopp

**Affiliations:** 1 Swiss Tropical and Public Health Institute, Kreuzstrasse 2, 4123 Allschwil, Switzerland; 2 University of Basel, Petersplatz 1, 4001 Basel, Switzerland; 3 Public Health Laboratory-Ivo de Carneri, Wawi, P.O. Box 122, Chake-Chake, Pemba United Republic of Tanzania; 4 Neglected Diseases Program, Zanzibar Ministry of Health, Social Welfare, Elderly, Gender and Children, P.O. Box 236, Unguja, United Republic of Tanzania

**Keywords:** Urogenital schistosomiasis, Control, Elimination, Intervention, Fine-scale mapping, Interruption of transmission, Micro-mapping, Precision mapping, Wayfinding, Zanzibar

## Abstract

**Background:**

Fine-scale mapping of schistosomiasis to guide micro-targeting of interventions will gain importance in elimination settings, where the heterogeneity of transmission is often pronounced. Novel mobile applications offer new opportunities for disease mapping. We provide a practical introduction and documentation of the strengths and shortcomings of GPS-based household identification and participant recruitment using tablet-based applications for fine-scale schistosomiasis mapping at sub-district level in a remote area in Pemba, Tanzania.

**Methods:**

A community-based household survey for urogenital schistosomiasis assessment was conducted from November 2020 until February 2021 in 20 small administrative areas in Pemba. For the survey, 1400 housing structures were prospectively and randomly selected from shapefile data. To identify pre-selected structures and collect survey-related data, field enumerators searched for the houses’ geolocation using the mobile applications Open Data Kit (ODK) and MAPS.ME. The number of inhabited and uninhabited structures, the median distance between the pre-selected and recorded locations, and the dropout rates due to non-participation or non-submission of urine samples of sufficient volume for schistosomiasis testing was assessed.

**Results:**

Among the 1400 randomly selected housing structures, 1396 (99.7%) were identified by the enumerators. The median distance between the pre-selected and recorded structures was 5.4 m. A total of 1098 (78.7%) were residential houses. Among them, 99 (9.0%) were dropped due to continuous absence of residents and 40 (3.6%) households refused to participate. In 797 (83.1%) among the 959 participating households, all eligible household members or all but one provided a urine sample of sufficient volume.

**Conclusions:**

The fine-scale mapping approach using a combination of ODK and an offline navigation application installed on tablet computers allows a very precise identification of housing structures. Dropouts due to non-residential housing structures, absence, non-participation and lack of urine need to be considered in survey designs. Our findings can guide the planning and implementation of future household-based mapping or longitudinal surveys and thus support micro-targeting and follow-up of interventions for schistosomiasis control and elimination in remote areas.

*Trial registration* ISRCTN, ISCRCTN91431493. Registered 11 February 2020, https://www.isrctn.com/ISRCTN91431493

**Supplementary Information:**

The online version contains supplementary material available at 10.1186/s40249-021-00928-y.

## Background

Schistosomiasis is among the 20 neglected tropical diseases (NTDs) defined by the World Health Organization (WHO) and is endemic in 78 countries worldwide [1–3]. The causative agent of schistosomiasis is a blood fluke of the genus *Schistosoma* that infects more than 200 million people worldwide [4]. The parasite is transmitted through skin contact with freshwater containing the infectious larval stages, which are released by an aquatic intermediate host snail vector [5]. Over the past decades, great progress was made in schistosomiasis control [6, 7]. In their new roadmap for neglected tropical diseases 2021–2030, the WHO set the global elimination of schistosomiasis as a public health problem and the validated absence of infection in humans in 25 among the 78 endemic countries as targets for 2030 [1].

The sustained implementation of control interventions, including mass drug administration (MDA) as the cornerstone, often supplemented with snail control, educational measures, or improvements in the socio-economic standard and access to clean water and sanitation, has resulted in decreasing schistosomiasis prevalences in many countries [8–11]. In areas where prevalences decline, the focality and heterogeneity of schistosomiasis becomes more pronounced [8, 12–14]. Typically, within a few years of annual or biannual MDA across districts and countries, many communities reach low schistosomiasis prevalence and infection intensity [10, 15, 16]. Some areas, however, remain as pockets of high transmission with persistent or reoccurring high prevalence levels despite intense interventions [8, 12–14, 17–20].

To achieve the WHO elimination goals, the pronounced spatial and temporal heterogeneity in schistosomiasis elimination settings will need to be considered in future intervention planning [8, 14, 20–23].

Fine-scale mapping of schistosomiasis is considered an essential requirement to move from morbidity control towards interruption of transmission in endemic areas [23]. Detailed fine-scale mapping of infection and disease patterns can help to identify low-risk and high-risk areas of transmission at sub-district level and to micro-target interventions in line with infection levels for optimal treatment and resource allocation [10, 14, 24, 25]. Android tablet-based applications containing or being able to connect to high-resolution maps to geolocate individuals’ residences in remote resource poor areas have been suggested as attractive approaches for fine-scale disease mapping [26, 27].

Here, we provide a practical introduction and documentation of the strengths and shortcomings of Global Position System (GPS)-based household identification and participant recruitment using Android tablet-based applications for fine-scale schistosomiasis mapping in a remote area in Pemba, United Republic of Tanzania.

## Methods

### Study area

**Fig. 1:**
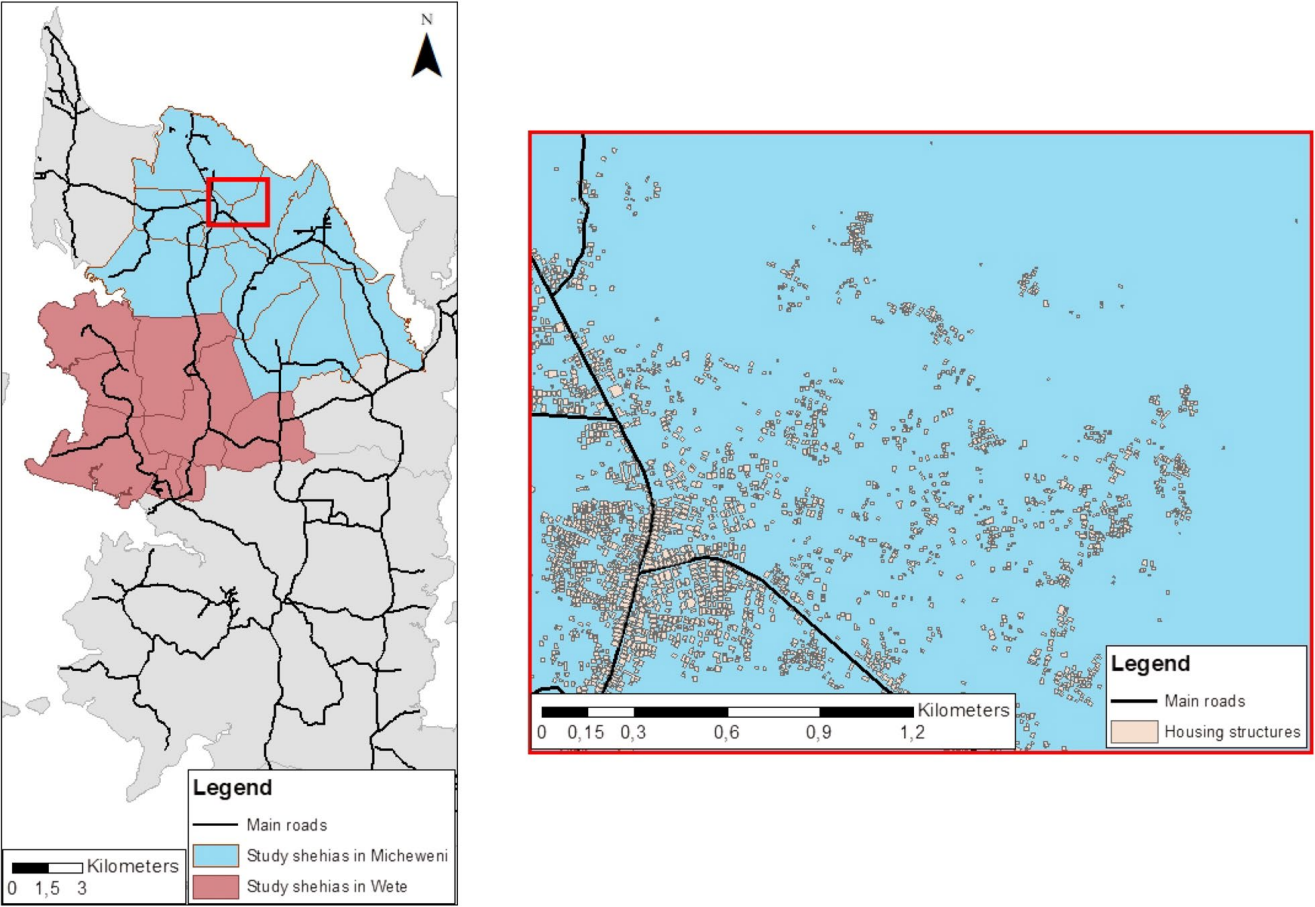
Study area of the SchistoBreak project in Pemba, Tanzania. The exemplary section shows the distribution of housing structures and main roads. The image base map (United Republic of Tanzania—Subnational administrative boundaries) was downloaded from OCHA services (https://data.humdata.org/dataset/tanzania-administrative-boundaries-level-1-to-3-regions-districts-and-wards-with-2012-population). The data source is: Tanzania National Bureau of Statistics/UN OCHA ROSA. The data are published under the following license: Creative Commons Attribution for Intergovernmental Organisations [CC BY-IGO; (https://creativecommons.org/licenses/by/3.0/igo/legalcode)]. Additionally, we received written permission to use and adapt the data from the UN Office for the Coordination of Humanitarian Affairs (OCHA). Additional shapefiles for the map (shehia boundaries and housing structures) were provided by the Zanzibar Commission for Lands to the Zanzibar Neglected Diseases Program

The fine-scale mapping approach described here is part of the cross-sectional baseline survey of the “SchistoBreak” study, a multi-year project that aims to investigate new tools and strategies for breaking schistosomiasis transmission [28]. Pemba and Unguja are the main islands of the Zanzibar Archipelago, a semi-autonomous part of the United Republic of Tanzania. Pemba, where our study is implemented, is divided into four districts: Micheweni, Wete, Chake Chake and Mkoani [29]. In total, the districts in Pemba contain 129 small administrative areas, which are called shehias [30]. The study area of the SchistoBreak project, where the here reported fine-scale mapping survey is part of, covers a total of 20 shehias in the north of Pemba. Among them, 12 shehias are located in Micheweni and eight in Wete district, respectively. The average population size in our study shehias was ~ 3900 individuals per shehia in 2012, with a minimum of ~ 2200 and a maximum of ~ 8600 inhabitants [31]. As indicated in Fig. 1, shehias in Pemba are mostly connected by tarmacked main roads. However, some rough road connections exist. Housing structures within the shehias are grouped in clusters, which are usually aligned along the main roads or located near unpaved streets or footpaths. Housing structures are mostly not characterized or identifiable by street names or house numbers. Urogenital schistosomiasis is endemic in the north of Pemba [32, 33]. Due to intense control and elimination efforts implemented across the Zanzibar islands over the past decades, the overall *S. haematobium* prevalence had reached a very low level of 3.4% in schoolchildren in 2020, constituted by many low-prevalence areas and a few remaining hotspot areas of transmission [8].

### Design of cross-sectional household survey

In the baseline survey of the SchistoBreak study, we aimed to assess the apparent prevalence of *S. haematobium* infections in the 20 study shehias in the north of Pemba. To gain a clear picture of the schistosomiasis distribution we conducted a cross-sectional community-based household survey from November 2020 until February 2021, based on randomly selected housing structures. For fine-scale mapping, we aimed to include 50 households with an average of five household members per shehia, and hence a total of 250 individuals per shehia. Details of the sample size calculations are provided in our published study protocol [28]. Eligible to participate in the household survey were all individuals aged 4 years or older living in the study shehias. Accounting for an estimated 30% dropout due to housing structures not being residential houses or refusal of household members to participate in the study, we randomized 70 housing structures per shehia. Our survey team consisted of seven field-enumerators. Each of them visited 10 housing structures per shehia. Shehias were visited for 3 subsequent days to allow enough time for revisiting houses in case the inhabitants were absent on Day 1 and/or for delayed urine submission on Day 2 or Day 3. Each enumerator was equipped with a tablet computer for navigation to their pre-selected housing structures using the offline navigation map application © 2020 MAPS.ME (https://www.maps.me/) and for electronic capturing of participant data using the data collection software © 2020 Open Data Kit (ODK; https://www.opendatakit.org/).

### Randomization of housing structures

For the randomization of houses, we used shapefile data of housing structures that were provided by the Zanzibar Commission for Lands to the Zanzibar Neglected Diseases Program. Shape file data could have also been obtained from © OpenStreetMap contributors (OSM). The OSM house shapefile data are published under the Open Database License (https://www.openstreetmap.org/copyright). Housing structures are indicated as polygons in shapefiles. For randomization of housing structures, for navigating to the randomized structures, and for conducting the questionnaire, we used the statistical software R version 3.5.1 (https://www.r-project.org/), the geospatial analysis software Esri ArcMap version 10.6.1, the offline map application MAPS.ME, and the data collection software ODK, respectively.

For randomization of the 70 housing structures, we first imported the shapefile polygon data into ArcMap and used its feature-to-point tool to calculate the centroid points of all housing polygons. As the information about the location of the centroids was provided in meters, we converted the meters into latitude and longitude coordinates as this is required by MAPS.ME. Subsequently, we exported the centroids given as shapefile data from ArcMap into R and randomly selected 70 houses per shehia.

### Stratification of housing structures per shehia and enumerator

After randomly selecting 70 houses per shehia in R, we imported the centroids of randomized housing structures back into ArcMap. With the selection-by-lasso tool of ArcMap, we stratified the centroids in equal-sized groups of 10 per each of the seven enumerators. Hereby, we took care that the 10 housing structures for each enumerator were as close in distance as possible to avoid the need for far-distance walking. We saved each group of housing structures per enumerator as a separate layer and shapefile and subsequently exported all single shapefiles to R. In R, we added a new variable “interviewer” and assigned a unique value to each group of housing structures per enumerator. With the new interviewer variable, we ensured to keep the group of housing structures in the next step, in which we combined all single shapefiles of housing groups into one large dataset. Subsequently, we saved the merged data containing the shehia name, the geolocation of all 70 randomized housing structures per shehia, and the group variable for seven groups with 10 housing structures each per shehia as csv-files and as shapefiles for display.

### Import of grouped housing structures per enumerator into ODK

Once the randomization process was completed, each housing structure was given a unique identifier code (with the variable name: "houseID"), which together with the shehia name, and latitude and longitude coordinates, was exported from the csv-files into an ODK excel sheet. In order to limit the choices displayed in ODK Collect to only those that belonged to the selected enumerator's group of housing structures, we used the “choice_filter” option of ODK. An example for using the choice_filter option with regards to the shehia and the enumerator is presented in Table 1, line 1–3 and Table 2, line 1–12. The example in Tables 1 and 2 shows a model for two shehias and two enumerators, each taking care of one group of housing structures per shehia.

**Table 1:** Customizing the ODK excel sheet for data collection and combined use with MAPS.ME: the survey sheet

line	type	name	label::english (en)	appearance	calculation	choice_filter
1	select_one interviewer	interviewer	Interviewer:			
2	select_one shehia	shehia	Shehia:			
3	select_one houseID	houseID	To which household are you going?			shehia = ${shehia} and interviewer = ${interviewer}
4	calculate	latitude			instance('houseID')/root/item[name = ${houseID}]/latitude	
5	calculate	longitude			instance('houseID')/root/item[name = ${houseID}]/longitude	
6	select_one modes	mode	How will you get there?			
7	integer	mapsme-wayfinding	Click to open maps.me	ex:com.mapswithme.maps.pro.action BUILD_ROUTE(lat_to = number(${latitude}), lon_to = number(${longitude}), router = ${mode})		
8	select_one modes	goodbye	Thanks!			latitude = 1 and longitude = 1

**Table 2:** Customizing the ODK excel sheet for data collection and combined use with MAPS.ME: the choices sheet

line	list_name	name	label::english (en)	shehia	interviewer	latitude	longitude
1	interviewer	interviewer 1	interviewer 1				
2	interviewer	interviewer 2	interviewer 2				
3	shehia	shehia 1	Shehia 1				
4	shehia	shehia 2	Shehia 2				
5	houseID	houseID-01	HouseID-01	shehia 1	interviewer 1	*.***** ^a^	**.*****
6	houseID	houseID-02	HouseID-02	shehia 1	interviewer 1	*.*****	**.*****
7	houseID	houseID-03	HouseID-03	shehia 1	interviewer 2	*.*****	**.*****
8	houseID	houseID-04	HouseID-04	shehia 1	interviewer 2	*.*****	**.*****
9	houseID	houseID-05	HouseID-05	shehia 2	interviewer 1	*.*****	**.*****
10	houseID	houseID-06	HouseID-06	shehia 2	interviewer 1	*.*****	**.*****
11	houseID	houseID-07	HouseID-07	shehia 2	interviewer 2	*.*****	**.*****
12	houseID	houseID-08	HouseID-08	shehia 2	interviewer 2	*.*****	**.*****
13	modes	pedestrian	Walking				
14	modes	vehicle	By bus or car				
15	modes	bicycle	By bicycle				

^a^ Geolocations are not shown to preserve confidentiality

### Combination of MAPS.ME and ODK for wayfinding to selected housing structures

The enumerators used the navigator app MAPS.ME to identify and find the way to each randomized housing structure in each shehia. We selected this app as it works offline and downloadable maps contain the housing structures and street data published on OSM. ODK and MAPS.ME apps were installed on the mobile devices (Samsung Galaxy Tab A tablets; Samsung Electronics, Seoul, South Korea) that were used by the enumerators. To connect ODK with MAPS.ME, we inserted into the ODK excel sheet the information presented in Table 1, line 3–8, and Table 2, line 5–15. As indicated in Table 1, line 3–5, the calculation of the geolocation is based on the houseID that is selected by the enumerator and presented in Table 2, line 5–12. Table 1, line 6 and the corresponding lines 13–15 in Table 2 serve to select different transport modes, e.g. walking, going by vehicle or by bicycle so that transport ways and transport time can be calculated by MAPS.ME. By opening the ODK questionnaire, choosing a houseID and selecting the transport mode on the terminal mobile device, a button saying “Click to open maps.me” appears as indicated in Table 1, line 7. By clicking the appearing button, the prior installed navigator app MAPS.ME opens and the geolocation of the selected houseID is automatically set as destination point and displays the way to go from the point of being to the housing structure of interest.

### Community-based data collection

To allow enough time for household identification, participant recruitment, questionnaire interview and urine collection, a shehia was visited by the field-enumerators for three subsequent days. On Day 1, most or all of the 10 pre-selected housing structures per enumerator were identified in the shehia community, and the geolocation and type of housing structure was recorded. In case the house was inhabited, household members were invited to participate in the study. Once a present adult household member agreed to participate, a questionnaire interview was conducted and instructions for urine collection from all household members aged ≥ 4 years were provided. One urine collection container per eligible household member was distributed and labelled with a unique picture (e.g. with a boat, star, cat or smiley) linked to the name of the participant recorded on a paper sheet to avoid confusion of urine samples. On Day 2, written informed consent forms for each participant, either signed by the participant or, in case of children, by their parent or legal guardian, were collected by the enumerator together with the urine samples from the participants. For all children 12–17 years old, an additional assent form signed by the adolescents themselves was also collected. Day 3 served as a mop-up day, when urine samples that were not submitted on Day 1 or Day 2 were collected.

### Data management and statistical analysis

All data were collected with ODK and transferred to the ODK Central Server hosted at Swiss Tropical and Public Health Institute (Swiss TPH). All data were cleaned and analyzed with R version 3.5.1. Anonymized data are available as Additional files 1, 2. To document the strengths and shortcomings of GPS-based household identification and participant recruitment for fine-scale schistosomiasis mapping in a remote area in Africa (and in our case Pemba island), we evaluated the results of the community-based household survey once the data collection was completed.

First, we calculated the number of identified housing structures that were no residential houses but other structures such as buildings under construction, schools or shops.

Second, to assess the concordance of the location of the pre-selected housing structure with the location of the housing structures identified in the shehia communities by the enumerator, we determined the median, minimum and maximum distance between the initially randomized centroids and the recorded geolocations across all shehias and per shehia.

Third, we determined the number of residential houses where inhabitants were not at home at any day of the visits or where all inhabitants refused to participate in the study. The dropout rate of housing structures was calculated by dividing the number of not included housing structures by the total number of initially selected housing structures.

Fourth, for households that agreed to participate in the study, we assessed the total number of household members per inhabited house and the number of individuals eligible to participate in the study. The dropout rate of individuals who were eligible but did not provide a urine sample of sufficient volume for schistosomiasis testing was calculated by dividing the number of participants who submitted a urine sample by the total number of eligible individuals, stratified by sex and age category (i.e. adults versus children, defined as individuals below the age of 18 years).

Finally, we determined the number of urine samples received from each household and calculated the percentage of households where all participants provided a urine sample of sufficient volume for testing.

## Results

### Housing structures and participating households

**Fig. 2:**
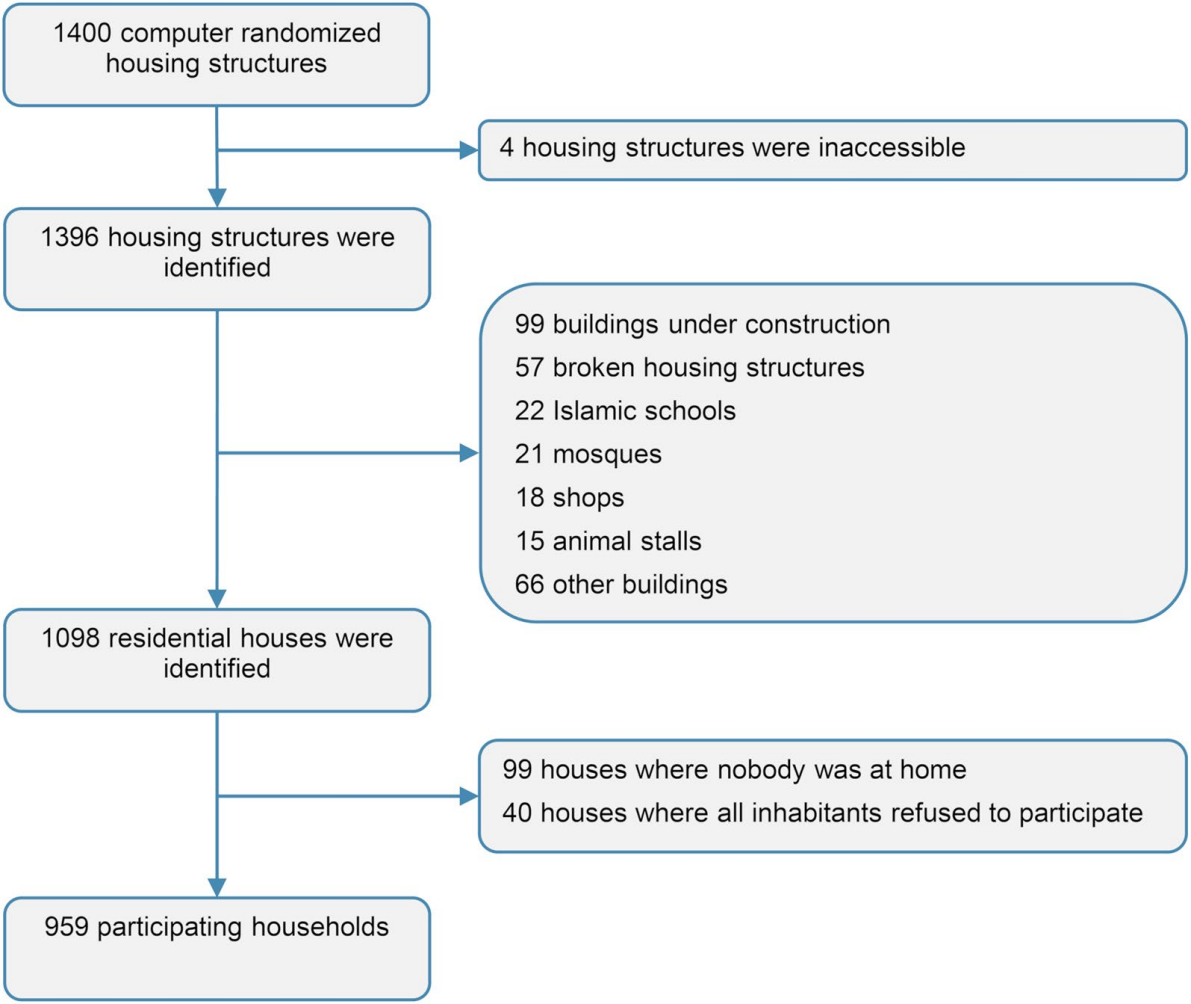
Housing structures and households participating in the fine-scale mapping survey in Pemba, Tanzania

Among the 1400 housing structures that were initially randomly selected to be surveyed in the 20 shehias, the enumerators were able to locate and identify 1396 housing structures (99.7%) (Fig. 2). Four housing structures were not identified due to inaccessibility. Among the 1396 housing structures correctly identified, 298 (21.3%) were no residential houses: 99 were buildings under constructions, 57 were broken housing structures, 22 were Islamic schools, 21 were mosques, 18 were shops, 15 were animal stalls and 66 were other buildings, such as public schools and offices. Among the 1098 residential houses, in 99 (9.0%) houses, inhabitants were absent during any of the visits of the enumerator and in 40 (3.6%) houses, inhabitants did not agree to participate in the household survey. Hence, the overall dropout rate of housing structures was 31.5% (441/1400).

### Distance between centroids and recorded geolocation of housing structures

**Fig. 3:**
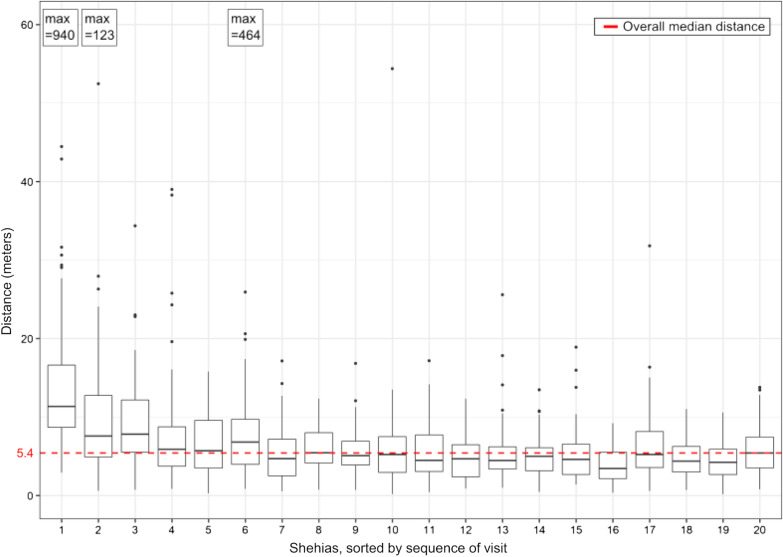
Distance between pre-selected and recorded geolocations of housing structures in the mapping survey in Pemba, Tanzania, per shehia. The horizontal middle lines in each boxplot indicate the median distance per shehia, the boxes indicate the lower and upper quartile, the vertical lines indicate the minimum and maximum excluding outliers, and the dots indicate outliers (more than 1.5 times the interquartile range below the first or above the 3rd quartile). For shehia 1, 2 and 6 the maximum outliers are indicated separately. The red dashed line indicates the median distance between pre-selected and recorded geolocations across all 20 shehias

As indicated in Fig. 3, the median distance between the randomized centroids of the housing structures and the geolocation recorded by the enumerator when arriving at the housing structure was 5.4 m (range: 0.2–940.3 m). While in the six shehias surveyed in the early study period (weeks 1–4) the median distance was 7.4 m (range: 0.3–940.3 m), in the 14 shehias surveyed in the later study period (week 4–12) the median distance was 4.8 m (range: 0.2–54.4 m).

**Fig. 4:**
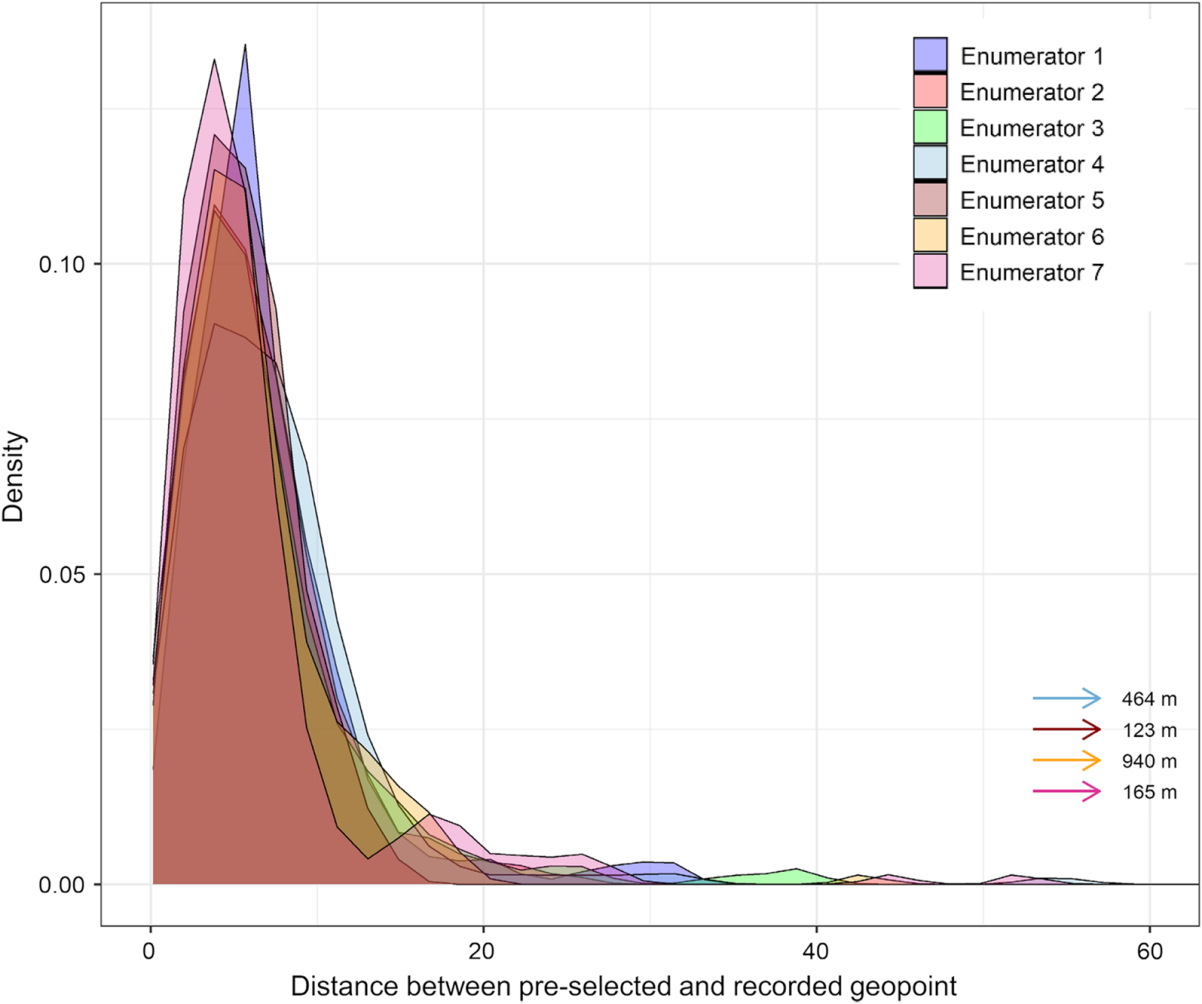
Distance between pre-selected and recorded geolocations of housing structures in the mapping survey in Pemba, Tanzania, per enumerator

Among the 1396 identified housing structures, four recorded geolocations were more than 100 m away from the pre-selected geopoints, taken by four different enumerators. In general, for all seven enumerators there was no major difference in the distribution of distances between the preselected and recorded geopoints (Fig. 4).

### Study participants

In total, 5340 individuals aged 0–99 years lived in the 959 participating households. In average, one household consisted of six individuals (range: 1–16 inhabitants).

Among the 5340 individuals, 4599 (86.1%) were eligible to participate in the study since their age was ≥ 4 years. Among them, 2324 (50.5%) were female and 2275 (49.5%) were male, and 2364 (51.4%) individuals were adults aged ≥ 18 years and 2233 (48.6%) were children aged 4–17 years. Two participants did not report their age. A total of 713 participants did not provide a urine sample, resulting in a dropout rate of 15.5% (713/4599). The dropout rate was higher in males (19.8%, 450/2275) than females (11.3%, 263/2323) and in adults (18.6%, 440/2364) compared with children (12.2%, 272/2233).

**Fig. 5:**
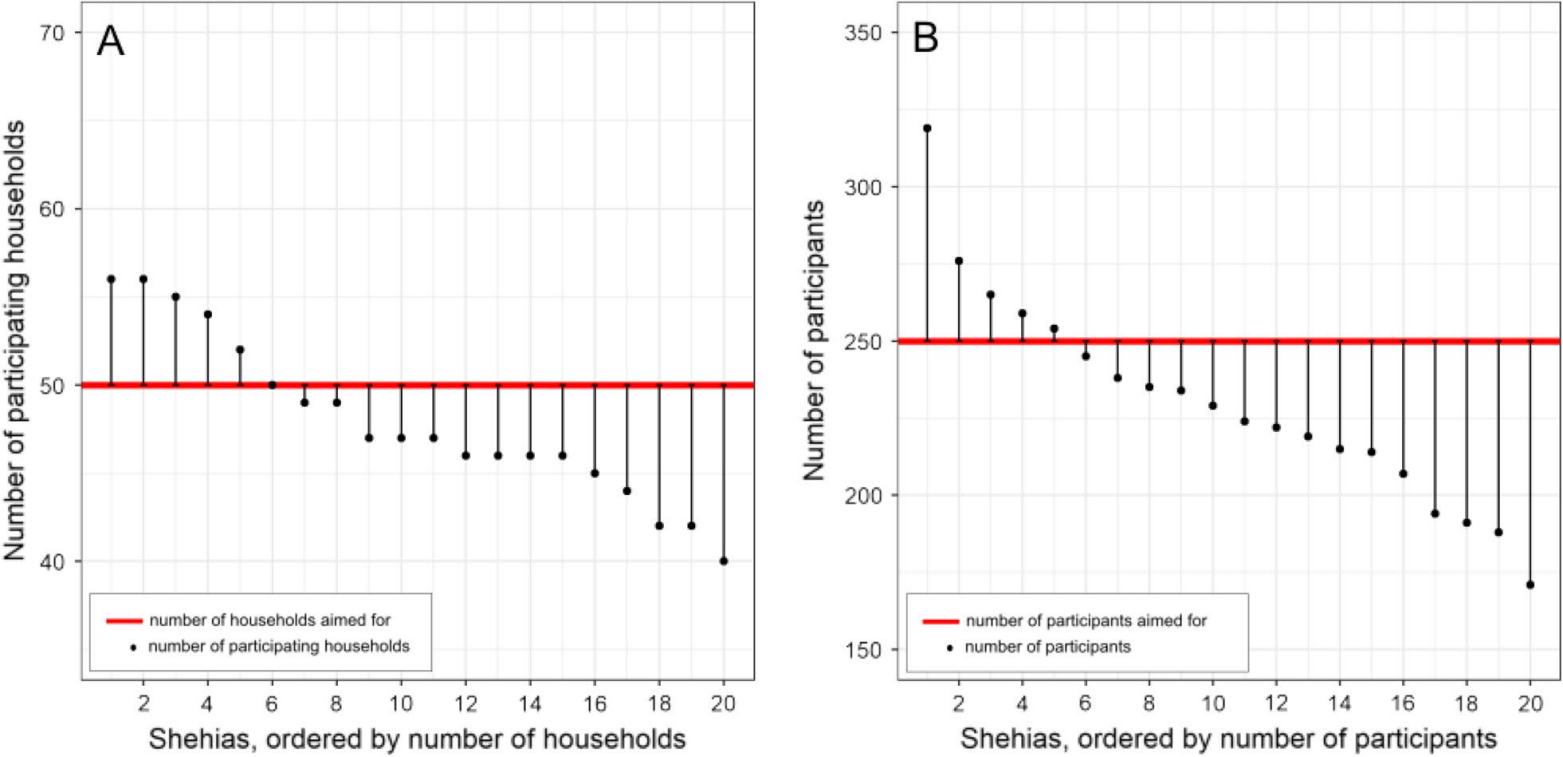
Number of households (**A**) and individuals (**B**) participating in the mapping survey in Pemba, Tanzania, per shehia

The number of participating households per shehia ranged from 40 to 56. In six among the 20 study shehias, the number of 50 households we aimed to include was achieved or exceeded (Fig. 5A). The number of participating individuals per shehia ranged from 171 to 319. In five shehias, the number of 250 participants we aimed for was achieved or exceeded (Fig. 5B).

**Fig. 6:**
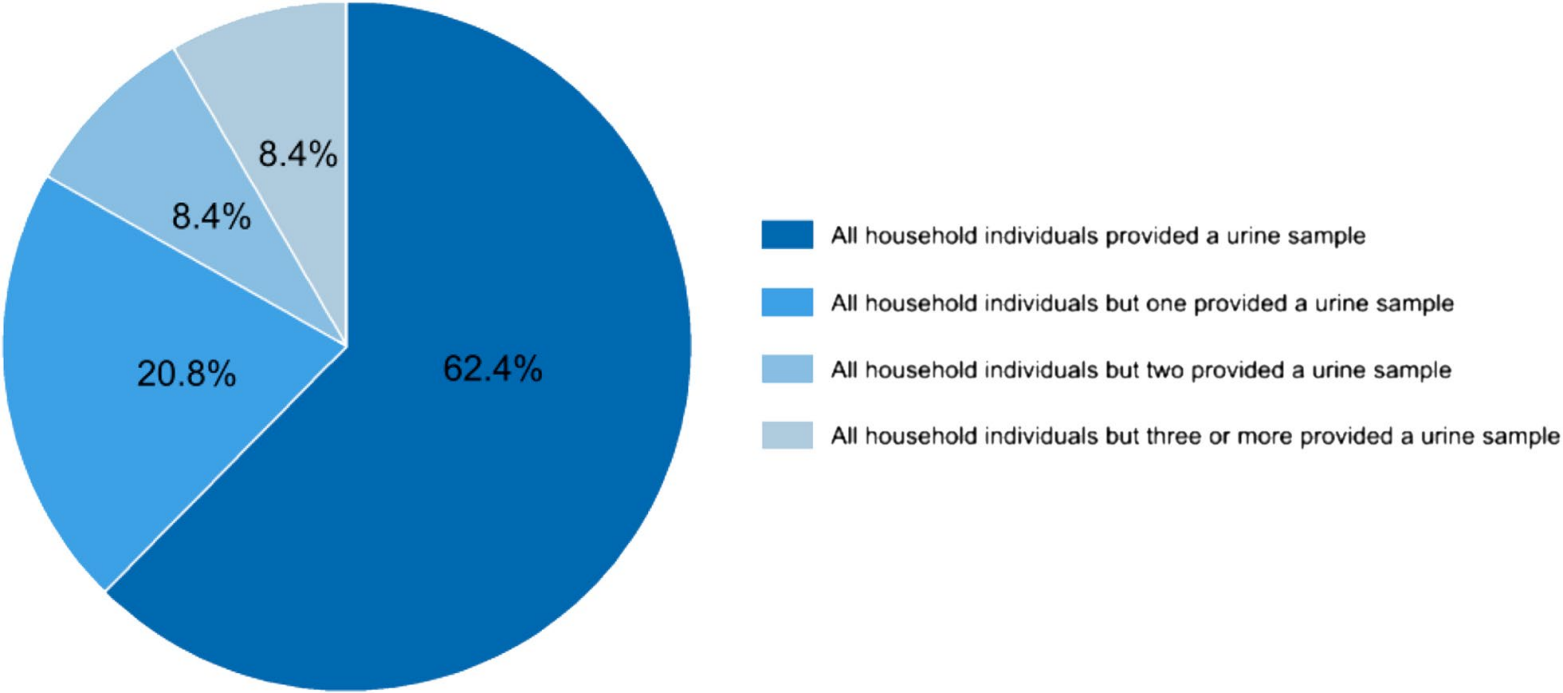
Provision of urine samples. Percentage of households with study participants providing a urine sample of sufficient volume (≥ 10 ml) for schistosomiasis testing in the fine-scale mapping survey of the SchistoBreak study in Pemba, Tanzania

Among the 959 participating households, in 598 (62.4%) households all eligible members provided a urine sample of sufficient volume (≥ 10 ml) for laboratory examinations, in 199 (20.8%) households all inhabitants but one individual provided a sufficiently large urine sample, in 81 (8.4%) households all inhabitants but two provided a sufficiently large urine sample, and in 81 (8.4%) households all inhabitants but three or more individuals provided a sufficiently large urine sample (Fig. 6).

## Discussion

In areas where schistosomiasis control progresses towards elimination, heterogeneity of transmission becomes more pronounced and fine-scale mapping of infection patterns is needed for effective micro-targeting of interventions at sub-district level [14, 23, 24, 28]. Novel tablet-based tools containing offline high-resolution maps offer new opportunities for disease mapping and household identification, particularly in remote areas where the distribution of housing structures do not follow an order such as street names or house numbers and where internet connectivity is poor [26].

Here, we aimed to provide a practical introduction and documentation of the strengths and shortcomings of tablet application-based household identification and participant recruitment for fine-scale schistosomiasis mapping in a rural area in Pemba, United Republic of Tanzania.

In our baseline mapping survey, almost all (99.7%) pre-selected housing structures were identified at the spot, in the communities, and located with a median distance of 5.4 m between the randomized centroids of the housing structures and the geolocation recorded by the enumerator. The computer-based randomization and offline navigation app-based geolocation of housing structures worked hence very well, even in remote villages in Pemba where “houses” may be small in size and located closely to each other and where internet connectivity is often limited or completely absent. The correct and exact identification of prospectively randomized housing structures is important for the accurate implementation of a community-based cross-sectional survey and also enables an easy follow-up of individuals or households for treatment or in longitudinal studies. Moreover, highly accurate small-scale spatial data can help to link household participant infection data with possible transmission sites of schistosomiasis, and hence to directly identify or model high-risk zones for infection and transmission that require special attention and targeted interventions. The location data can also be used to assess the distance to the next primary health care unit. A study conducted in rural Zambia showed that the preciseness of geographical cluster locations is crucial to link individuals to the correct health provider, such as primary health care units [34].

The dropout rate due to non-residential housing structures in our survey was 21.3%, absence of inhabitants was 9.0%, refused participation of households was 3.6%, and inability to provide a sufficiently large urine sample among all study participants was 15.5%. A very similar dropout rate of 24.1% due to uninhabited houses was observed in a household study based on spatial randomization from rural and urban regions of Cameroon [35]. The absence of inhabitants in our study was, however, considerably lower than in a comparable GPS-based household survey conducted in Haiti where the absence of inhabitants was 16.4% [36]. This lower rate in our study might be explained by the fact that our enumerators visited the residential houses in a shehia for a second day if no individuals were at home the first day. The refusal dropout rate of 3.6% was, however, the same in our study as in the GPS-based household study conducted in Haiti [36]. A considerably higher dropout rate of 29.0% due to absence and/or inability to provide a urine and stool sample was observed in a household survey in Yemen, which was conducted to assess the local schistosomiasis prevalence [37]. A reason for the higher dropout in this study might be that not only urine but also stool samples were collected, which is more embarrassing to produce, collect and submit. In summary, our results show that our originally estimated overall dropout rate of 30% due to housing structures not being residential houses or refusal of household members to participate in the study (versus the actually observed dropout rate of 31.5%) was realistic and can serve as a guiding value for future survey design planning and sample size calculations, provided that 2 or 3 days are planned for participant recruitment and sample collection. In case households are only visited on a single day, higher dropout rates need to be considered in schistosomiasis mapping surveys, due to the potential absence of household members, or their inability or unwillingness to provide a urine or stool sample straight away. This being said, for *S. haematobium* diagnosis, urine samples are ideally collected in the late morning or early afternoon, when egg excretion is highest [38]. If urine samples can be produced and kept for the enumerator at any time of the day or night, this might impact on the diagnostic results and hamper accurate disease mapping.

With our practical introduction to and documentation of geolocation-based household identification and participant recruitment, we provide a rigorous basis for future survey planning. Particular strengths of our suggested approach are that it is low-cost, precise and easy to implement in the field as the final ODK questionnaire on the mobile device includes an automated link to the offline navigation system MAPS.ME. Due to the fully offline procedure it can also be used in remote areas without access to stable internet connections. Second, if data of housing structures are available on OSM, the fine-scale mapping approach is easy to replicate in other countries and for other purposes, particularly with the hands-on explanation provided in this article.

However, there are also important limitations. First, the approach relies on the availability of up-to-date (housing) data uploaded on OSM. In Pemba, new housing structures appear and disappear within a short time, or might be “under construction” for several years as has also been observed in other environments [36]. Without current data that include all new housing structures, individuals living in these housing structures will not be included in the randomization and missed for disease mapping. Without current data that mark or deleted demolished housing structures, the dropout rate will increase. To complement a higher dropout of housing structures if such invalid housing structures still appear in the data, the sample size could be increased or replacement-housing structures could be included in the survey, as done in other studies [35, 39]. Second, our approach relies on the accessibility of the housing structures. For example, four housing structures could not be included in our survey due to inaccessibility, mainly as they were located in a military area. The data that provided the basis for the randomization of housing structures did not show the military area and therefore the area was not excluded prior to randomization. Hence, the success of our mapping approach is very much depending on the availability of up-to-date high-resolution data, clearly indicating zones with restricted access.

## Conclusions

We show that the fine-scale mapping approach with the combination of ODK and an offline navigation application installed on tablet computers allows a very precise identification of pre-randomized housing structures. Dropouts due to non-residential houses, absence, non-participation and lack of urine need to be considered in sample size calculations. Our results can guide the planning and implementation of future household-based mapping or longitudinal surveys and thus support micro-targeting and follow-up of interventions for schistosomiasis control and elimination in remote areas.

## Supplementary Information

**Additional file 1.** Supplementary data that support the findings of this study from Nov 2020 until Feb 2021.

**Additional file 2. ** Supplementary data dictionary that explains the data collected from Nov 2020 until Feb 2021.

## Data Availability

Most relevant data are within the manuscript and its additional information files. Geopoints of the housing structures’ centroids and geolocations recorded by the field enumerators cannot be shared to preserve spatial confidentiality.

## Abbreviations

EKNZ: Ethikkommission Nordwest- und Zentralschweiz
MDA: Mass drug administration
NTD: Neglected tropical disease
ODK: Open Data Kit
OSM: Open Street Map
Swiss TPH: Swiss Tropical and Public Health Institute
WHO: World Health Organization
ZAHRI: Zanzibar Health Research Institute
